# Which Comprehensive Geriatric Assessment (CGA) instruments are currently used in Germany: a survey

**DOI:** 10.1186/s12877-024-04913-6

**Published:** 2024-04-17

**Authors:** Jennifer Kudelka, Malte Ollenschläger, Richard Dodel, Bjoern M. Eskofier, Markus A. Hobert, Klaus Jahn, Jochen Klucken, Bendix Labeit, M. Cristina Polidori, Tino Prell, Tobias Warnecke, Christine A. F. von Arnim, Walter Maetzler, Andreas H. Jacobs, for the DGG working group Neurology, Marija Djukic, Ralf-Peter Häussermann, Marek Jauss, Sandra Schütze, Cornelius J. Werner

**Affiliations:** 1 Department of Neurology, University Hospital Schleswig-Holstein, Arnold-Heller-Straße 3, Kiel, 24105 Germany; 2 Department of Artificial Intelligence in Biomedical Engineering (AIBE), Machine Learning and Data Analytics Lab, Friedrich-Alexander-Universität Erlangen-Nürnberg (FAU), Erlangen, Germany; 3 Department of Molecular Neurology, University Hospital Erlangen, Erlangen, Germany; 4 Chair of Geriatric Medicine, University Duisburg-Essen, Essen, Germany; 5 Schön Klinik Bad Aibling, Neurology and Geriatrics, Bad Aibling, Germany; 6 German Center for Vertigo and Balance Disorders (DSGZ), Ludwig-Maximilians University (LMU) of Munich, Munich, Germany; 7 Luxembourg Centre for Systems Biomedicine (LCSB), University of Luxembourg, Esch-Sur-Alzette, Luxembourg; 8 Luxembourg Institute of Health (LIH), Strassen, Luxembourg; 9 Centre Hospitalier de Luxembourg (CHL), Luxembourg, Luxembourg; 10 Department of Neurology With Institute of Translational Neurology, University Hospital Münster, Münster, Germany; 11 Ageing Clinical Research, Department II of Internal Medicine and Center for Molecular Medicine Cologne, University of Cologne, Faculty of Medicine and University Hospital Cologne, Cologne, Germany; 12 CECAD, University of Cologne, Faculty of Medicine and University Hospital Cologne, Cologne, Germany; 13 Department of Geriatrics, Halle University Hospital, Halle (Saale), Germany; 14 Department of Neurology and Neurorehabilitation, Klinikum Osnabrueck – Academic teaching hospital of the University of Muenster, Osnabrueck, Germany; 15 Department of Geriatrics, University of Göttingen Medical Center, Göttingen, Germany; 16 Department of Geriatrics & Neurology, Johanniter Hospital Bonn, Johanniter Strasse 1-3, Bonn, 53113 Germany; 17 Centre for Integrated Oncology (CIO) of the University of Bonn, Bonn, Germany; 18 European Institute for Molecular Imaging (EIMI) of the Westfälische Wilhelms University (WWU), Münster, Germany

**Keywords:** Comprehensive geriatric assessment, CGA, Frailty, Activities of daily living, Self-help capability, Cognition, Depression, Delirium, Comorbidities, Dysphagia

## Abstract

**Background:**

The Comprehensive Geriatric Assessment (CGA) records geriatric syndromes in a standardized manner, allowing individualized treatment tailored to the patient’s needs and resources. Its use has shown a beneficial effect on the functional outcome and survival of geriatric patients. A recently published German S1 guideline for level 2 CGA provides recommendations for the use of a broad variety of different assessment instruments for each geriatric syndrome. However, the actual use of assessment instruments in routine geriatric clinical practice and its consistency with the guideline and the current state of literature has not been investigated to date.

**Methods:**

An online survey was developed by an expert group of geriatricians and sent to all licenced geriatricians (*n* = 569) within Germany. The survey included the following geriatric syndromes: motor function and self-help capability, cognition, depression, pain, dysphagia and nutrition, social status and comorbidity, pressure ulcers, language and speech, delirium, and frailty. Respondents were asked to report which geriatric assessment instruments are used to assess the respective syndromes.

**Results:**

A total of 122 clinicians participated in the survey (response rate: 21%); after data cleaning, 76 data sets remained for analysis. All participants regularly used assessment instruments in the following categories: motor function, self-help capability, cognition, depression, and pain. The most frequently used instruments in these categories were the Timed Up and Go (TUG), the Barthel Index (BI), the Mini Mental State Examination (MMSE), the Geriatric Depression Scale (GDS), and the Visual Analogue Scale (VAS). Limited or heterogenous assessments are used in the following categories: delirium, frailty and social status.

**Conclusions:**

Our results show that the assessment of motor function, self-help capability, cognition, depression, pain, and dysphagia and nutrition is consistent with the recommendations of the S1 guideline for level 2 CGA. Instruments recommended for more frequent use include the Short Physical Performance Battery (SPPB), the Montreal Cognitive Assessment (MoCA), and the WHO-5 (depression). There is a particular need for standardized assessment of delirium, frailty and social status. The harmonization of assessment instruments throughout geriatric departments shall enable more effective treatment and prevention of age-related diseases and syndromes.

## Background

The currently growing and ageing population leads to an increasing proportion of patients with multimorbidity and functional impairment. This is not only a medical challenge, but also an increasing economic burden for the health care system [1, 2]. Consequently, geriatrics as a multidisciplinary medical specialty is becoming increasingly important [3]. The special feature of geriatric medicine is the treatment of patients with multimorbidity, who have limitations in various functional domains such as motor function, cognition, mood, and continence, but also self-help capability, the ability to swallow, and pain-related alterations. The geriatric approach is not exclusively disease-oriented and focuses especially on functional status. Therefore, geriatric patients require a multidimensional therapeutic approach that covers all domains of the International Classification of Functioning, Disability and Health (ICF) model, including psychosocial factors such as daily activities and participation [4]. Although their negative impact on patients' quality of life and social participation is well established, geriatric syndromes are often underdiagnosed in clinical practice as they are not based on a single cause-effect mechanism, but often on dysfunctionalities in multiple organ systems [5].

The comprehensive geriatric assessment (CGA) was developed to „determine an older person’s medical, psychosocial, functional, and environmental resources and problems “ [6] in a standardized manner. In Germany, the CGA distinguishes between three levels of assessment: Level 1 is used to identify a geriatric patient. Level 2 serves as a basic assessment, which is a mandatory requirement for standardized early rehabilitative geriatric treatment in Germany. Level 3 is used to differentiate health problems more precisely, especially if the previous levels have revealed signs of impairment [4]. Based on the CGA, a multidisciplinary team will make treatment decisions that are tailored to the individual patient and include all aspects of life, while establishing a benchmark for long-term follow-up [7]. Clinical implementation of CGA has demonstrated its beneficial effects on functional status and survival of geriatric patients in acute and subacute settings in several randomized controlled trials [7–9].

However, a broad variety of assessment instruments exists for each geriatric syndrome [10], which poses a challenge in deciding which specific assessment instrument to use in daily practice. Therefore, a S1 guideline for CGA level 2 has been established in Germany to guide the decision-making process [4]. In Germany, there are four levels of guidelines (S1, S2e, S2k, S3), with the S1 guideline representing the lowest level [11]. This guideline aims to provide differentiated recommendations for the use of assessment instruments in CGA. In order to work efficiently in the geriatric context, assessment instruments must be both patient- and resource-friendly, provide quantitative data at diagnosis and follow-up, and thus constitute the basis for treatment decisions, efficacy assessment and prognosis.

Apart from the clinical context, the selection of assessment instruments has an impact on the conduct of clinical trials and studies. For example, in the field of chronic diseases, such as Parkinson's disease, there is a wide range of assessment instruments used in cohort studies. This results in a reduced comparability of the collected data and hinders the harmonization of data sets [12]. This situation reveals an obligation to ensure comparability of collected data in both clinical and scientific settings in order to minimize burden on geriatric patients and ensure optimal treatment decisions.

To assess the degree of standardization and the need for future harmonization, this study aims to investigate which CGA instruments are currently used in the various indications on geriatric wards. It is further assessed to what extent the current status quo corresponds to the recommendations of the recently published S1 guideline for level 2 CGA.

## Methods

### Survey development

In cooperation with the working group Neurology of the German Geriatrics Society (*Deutsche Gesellschaft für Geriatrie*, DGG) and with the Department of Molecular Neurology, University Hospital Erlangen as well as the Machine Learning and Data Analytics Lab of the Friedrich-Alexander-University Erlangen-Nuremberg (FAU), an online survey on the use of CGA instruments was created.

The content of the questionnaire was developed by the task force Neurogeriatrics, an expert group of geriatricians and neurologists in clinical leading roles in geriatric hospitals in Germany. The aim of the questionnaire was to evaluate the most commonly used assessment instruments for a broad spectrum of (neuro)geriatric syndromes. It should be pointed out that in German hospitals, geriatric assessment is required in at least five domains (functional, social) from health insurance companies in the early rehabilitative treatment of geriatric patients.

Participants were asked what assessment instruments they use in a standardized manner on their geriatric wards to evaluate the following syndromes: motor function and self-help capability, cognition, depression, pain, dysphagia and nutrition, social status and comorbidity, pressure ulcers, language and speech, delirium, and frailty. For each geriatric syndrome, a number of assessment instruments were predefined that, in the experience of the task force Neurogeriatrics and according to the S1 guideline for level 2 CGA [4], are frequently used to assess the particular syndrome. Moreover, participants could specify additional instruments in a free text area. Furthermore, participants were asked which assessment instrument they use on which occasion. The following selection options were available:

- standardized on admission
- standardized before discharge
- standardized during inpatient treatment
- standardized as post/progression/follow-up after inpatient treatment or on readmission
- in the context of specific treatments/diagnoses.

### Participant acquisition

The survey was announced to all licensed geriatricians within Germany by email contact. Subsequently, access data to the online survey, consisting of a participant code for legitimation, were sent to the participants. The legitimation code was furthermore used for pseudonymization. The decoding between e-mail addresses and legitimation code is exclusively stored at the DGG to maintain the possibility to delete data sets upon request of participants. Other parties involved in the analysis of the data set did not have access to the e-mail addresses at any point.

Participants were provided with information about the study before starting the survey. Consent was implied from their voluntary participation in the survey. Participants were informed that they can revoke their participation at any time and request deletion of the submitted data.

The contacting was performed in two steps: In a first step (February 2021), *n* = 569 included geriatricians were contacted, of whom *n* = 43 participated in the survey. After twelve weeks, a further query was carried out, in which *n* = 390 participants were contacted again and *n* = 79 took part. This resulted in a total number of *n* = 122 participants. Participants spent a mean of 23.5 min to complete the survey.

### Data processing

**Fig. 1:**
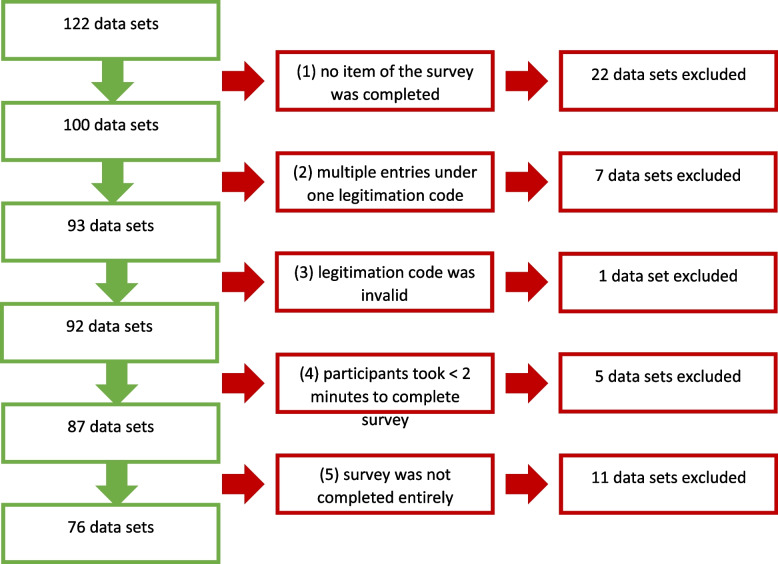
Cleaning process of the collected data sets

Data from the *n* = 122 participants were submitted to a cleaning process in which datasets were excluded if (1) no item of the survey was completed, (2) multiple entries were made under one legitimation code, (3) the legitimation code was invalid, (4) participants took less than two minutes to complete the survey, or (5) participants did not complete the survey entirely. A flow-chart of the cleaning process is displayed in Fig. 1. After the cleaning process, 76 data sets remained to be included in the analysis. The analysis was performed quantitatively and descriptively by the Chair of Computer Science at FAU.

### Quantification

The percentage of participants, who performed a specific assessment parameter was determined at the time points mentioned above during the hospital stay. Quantitative parameters were expressed as absolute numbers or in percentage of participating centers, respectively.

## Results

**Fig. 2:**
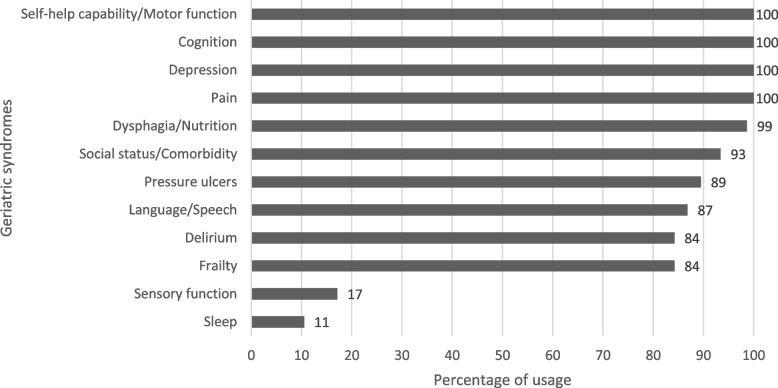
Percentage of usage of assessment instruments for each syndrome

The results are presented below by category in the order of overall percentage of usage (see Fig. 2). Overall, only results from categories that were used by the majority of participants (> 80%) are presented. All participants (*n* = 76, 100%) used at least one assessment instrument in the following categories: motor function, self-help capability, cognition, depression, and pain. Few participants indicated regularly using assessment instruments to record sensory function (*n* = 13, 17.1%) and sleep (*n* = 8, 10.5%). Thus, these categories are not presented in detail.

### Motor function

**Fig. 3:**
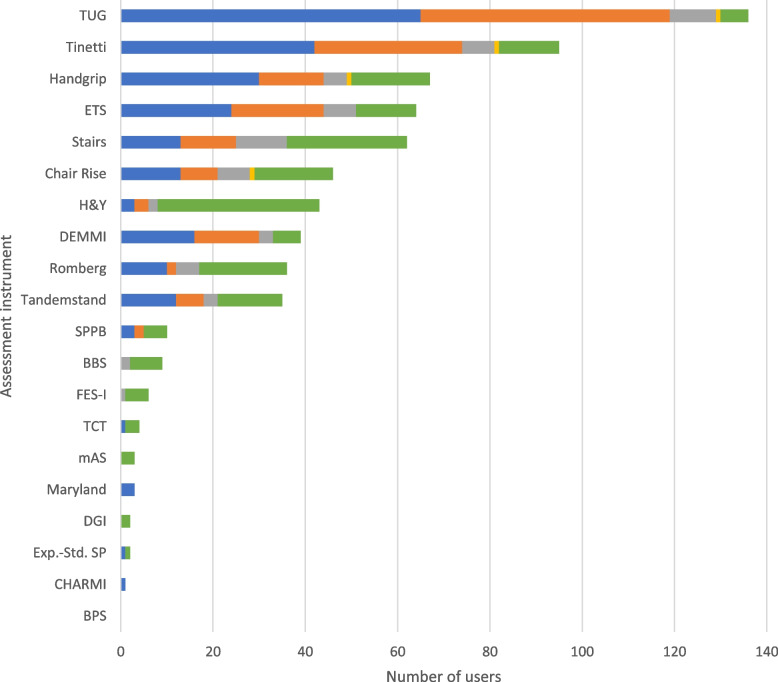
Motor function assessment instruments. The use of the assessment instruments at the color-coded time points is presented in absolute numbers (blue = standardized on admission; orange = standardized before discharge; gray = standardized during inpatient treatment; yellow = standardized as post/progression/follow-up after inpatient treatment or on readmission; green = in the context of specific treatments/diagnoses). Multiple responses were possible. BBS = Berg-Balance-Scale, BPS = Back Performance Scale, CHARMI = *Charité Mobilitäts-Index*, DEMMI = De Morton Mobility Index, DGI = Dynamic Gait Index, ETS = *Esslinger Transferskala*, Exp.-Std. SP = *Expertenstandard Sturzprophylaxe*, FES-I = Falls Efficacy Scale – International, H&Y = Hoehn & Yahr stages, Maryland = *Sturzrisiko nach Maryland*, mAS = modified Ashworth Scale, SPPB = Short Physical Performance Battery, TCT = Trunk Control Test, TUG = Timed-up-and-go

A variety of different assessment instruments were reported to be used on various occasions. Of all assessment instruments, the Timed Up and Go (TUG; [13]) was used most frequently both on admission (*n* = 65, 85.5%) and before discharge (*n* = 54, 71.1%). Other assessment instruments frequently used on admission were the Tinetti test [14] (*n* = 42, 55.3%), grip strength (*n* = 30, 39.5%), the *Esslinger Transferskala* (ETS; [15]) (*n* = 24, 31.6%) and the De Morton Mobility Index (DEMMI; [16]) (*n* = 16, 21.1%). By contrast, the Hoehn & Yahr stage [17] (*n* = 35, 46.1%) and stair climbing (*n* = 26, 34.2%) were more frequently used in context of specific treatments and diagnoses. Results are presented in Fig. 3.

### Self-help capability

**Fig. 4:**
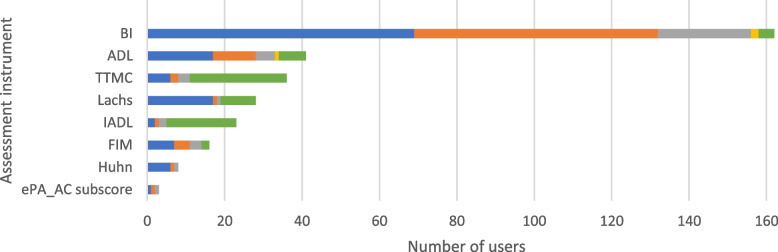
Self-help capability assessment instruments. The use of the assessment instruments at the color-coded time points is presented in absolute numbers (blue = standardized on admission; orange = standardized before discharge; gray = standardized during inpatient treatment; yellow = standardized as post/progression/follow-up after inpatient treatment or on readmission; green = in the context of specific treatments/diagnoses). Multiple responses were possible. ADL = activities of daily living, BI = Barthel index, ePA-AC = *ergebnisorientiertes PflegeAssessment Acute Care©*, FIM = Functional Independence Measure, Huhn = *Sturzrisiko nach Huhn*, IADL = instrumental activities of daily living, TTMC = Timed Test of Money Counting

The most commonly used instrument to assess self-help capability on admission (*n* = 69, 90.8%) and before discharge (*n* = 63, 82.9%) was the Barthel index (BI; [18]). A minority of participants reported to use the activities of daily living (ADL; [19]) (*n* = 17, 22.4%) and the Lachs screening [20] (*n* = 17, 22.4%) on admission. The Timed Test of Money Counting (TTMC; [21]) (*n* = 25, 32.9%) and the instrumental activities of daily living (IADL; [22]) (*n* = 18, 23.7%) were used more frequently in the context of specific treatments and diagnoses. Results are presented in Fig. 4.

### Cognition

**Fig. 5:**
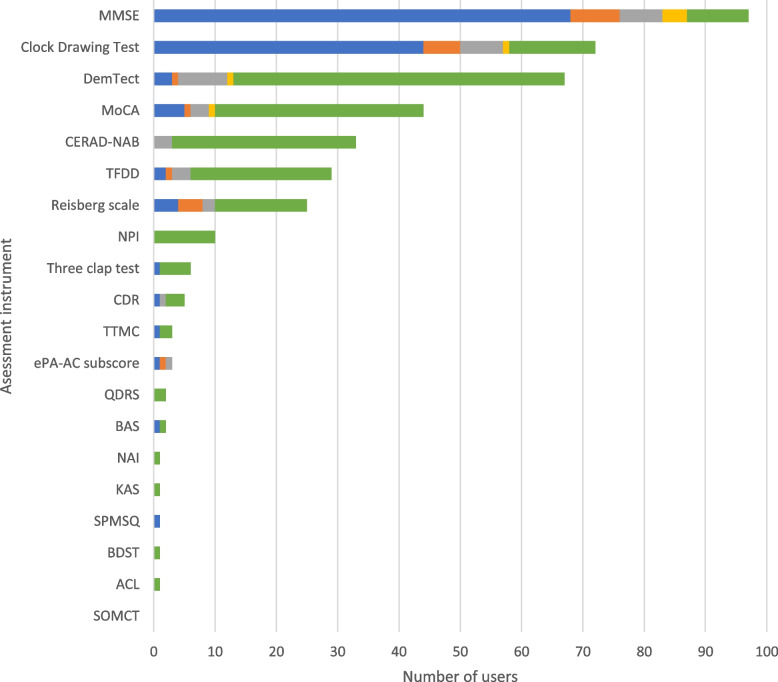
Cognition assessment instruments. The use of the assessment instruments at the color-coded time points is presented in absolute numbers (blue = standardized on admission; orange = standardized before discharge; gray = standardized during inpatient treatment; yellow = standardized as post/progression/follow-up after inpatient treatment or on readmission; green = in the context of specific treatments/diagnoses). Multiple responses were possible. ACL = Allen Cognitive Level, BAS = Brief Alzheimer Screen, BDST = *Bamberger Demenz Screening Test*, CDR = Clinical Dementia Rating Questionnaire, CERAD-NAB = Consortium to Establish a Registry on Alzheimer’s Disease—Neuropsychological Assessment Battery, DemTect = *Demenz-Detektion*, ePA-AC = *ergebnisorientiertes PflegeAssessment Acute Care©*, KAS = *Kölner Apraxie-Screening*, MMSE = Mini Mental State Examination, MoCA = Montreal Cognitive Assessment, NAI = *Nürnberger Altersinventar*, NPI = *Neuropsychiatrisches Inventar*, QDRS = Quick Dementia Rating System, SOMCT = Short Orientation Memory Concentration Test, SPMSQ = Short Portable mental Status Questionnaire, TFDD = *Test zur Früherkennung von Demenz mit Depressionsabgrenzung*, TTMC = Timed Test of Money Counting

Two cognition screening assessments were reported to be collected most frequently at the time of admission: The Mini Mental State Examination (MMSE; [23]) (*n* = 68, 89.5%) and the Clock Drawing Test (CDT) (*n* = 44, 57.9%). Cognition tests that were often performed in specific situations are the *Demenz-Detektion* (DemTect; [24]) (*n* = 54, 71.1%), the Montreal Cognitive Assessment (MoCA; [25]) (*n* = 34, 44.7%), the Consortium to Establish a Registry on Alzheimer’s Disease—Neuropsychological Assessment Battery (CERAD-NAB; [26]) (*n* = 27, 35.5%) and the *Test zur Früherkennung von Demenz mit Depressionsabgrenzung* (TFDD; [27]) (*n* = 23, 30.3%). Results are presented in Fig. 5.

### Depression

**Fig. 6:**
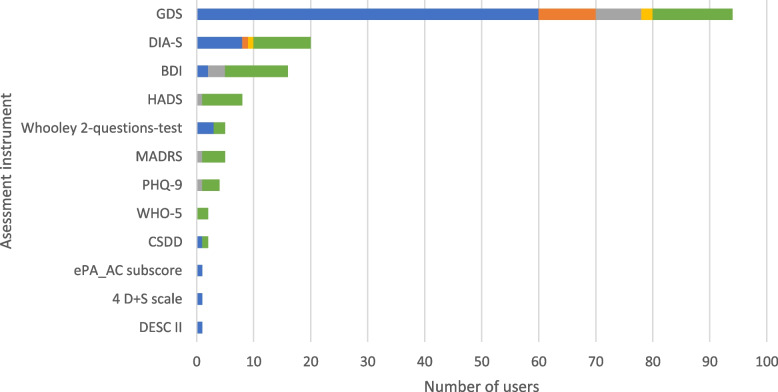
Depression assessment instruments. The use of the assessment instruments at the color-coded time points is presented in absolute numbers (blue = standardized on admission; orange = standardized before discharge; gray = standardized during inpatient treatment; yellow = standardized as post/progression/follow-up after inpatient treatment or on readmission; green = in the context of specific treatments/diagnoses). Multiple responses were possible. BDI = Beck Depression Inventory, CSDD = Cornell Scale for Depression in Dementia, DESC = *Rasch-basierte Depressionsscreening*, DIA-S = *Depression im Alter-Skala*, ePA-AC = *ergebnisorientiertes PflegeAssessment Acute Care©*, GDS = Geriatric Depression Scale, HADS = Hospital Anxiety and Depression Scale, MADRS = Montgomery-Asberg Depression Rating Scale, PHQ = Patient Health Questionnaire, WHO-5 = WHO-Five Well-Being Index

The most frequently used assessment instrument on admission was the Geriatric Depression Scale (GDS; [28]) (*n* = 59, 77.6%). Other assessment instruments used by some participants were the *Depression-im-Alter Skala* (DIA-S; [29]) (admission: *n* = 8, 10.5%; specific situations: *n* = 10, 13.2%), and, in specific situations, the Beck Depression Inventory (BDI; [30]) (*n* = 11, 14,5%), the Hospital Anxiety and Depression Scale (HADS; [31]) (*n* = 7, 9.2%) and the World Health Organization-Five Well-Being Index (WHO-5; [32]) (*n* = 2, 2.6%). Results are presented in Fig. 6.

### Pain

**Fig. 7:**
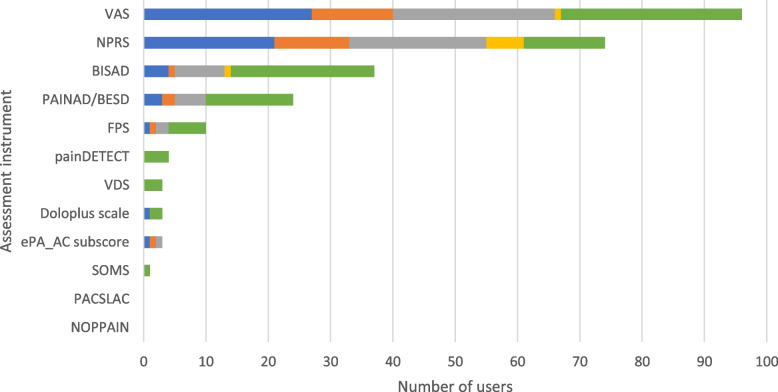
Pain assessment instruments. The use of the assessment instruments at the color-coded time points is presented in absolute numbers (blue = standardized on admission; orange = standardized before discharge; gray = standardized during inpatient treatment; yellow = standardized as post/progression/follow-up after inpatient treatment or on readmission; green = in the context of specific treatments/diagnoses). Multiple responses were possible. BESD = *Beurteilung von Schmerzen bei Demenz*, BISAD = *Beobachtungsinstrument für das Schmerzassessment bei alten Menschen mit Demenz*, ePA-AC = ergebnisorientiertes PflegeAssessment Acute Care©, FPS = Faces Pain Scale, NOPPAIN = Non Communicative Pain Assessment Instrument, NPRS = Numeric Pain Rating Scale, PACSLAC = Pain Assessment Checklist for Sensiors with Limited Ability to Communicate, PAINAD = Pain Assessment in Advanced Dementia Scale, SOMS = *Screening für somatoforme Störungen*, VAS = Visual Analogue Scale, VDS = Verbal Descriptor Pain Scale

Two assessment instruments were used most frequently on admission: The Visual Analogue Scale (VAS) (*n* = 27, 35.5%) and the Numeric Pain Rating Scale (NPRS) (*n* = 21, 27.6%). Other assessment instruments were mostly used in specific situations, e.g. the *Beobachtungsinstrument für das Schmerzassessment bei alten Menschen mit Demenz* (BISAD; [33]) (*n* = 21, 27.6%), the Pain Assessment in Advanced Dementia Scale (PAINAD; [34]) (*n* = 14, 18.4%), the Faces Pain Scale (FPS; e.g. [35]) (*n* = 6, 7.9%), or the painDETECT [36] (*n* = 4, 5.3%). Results are presented in Fig. 7.

### Dysphagia and nutrition

**Fig. 8:**
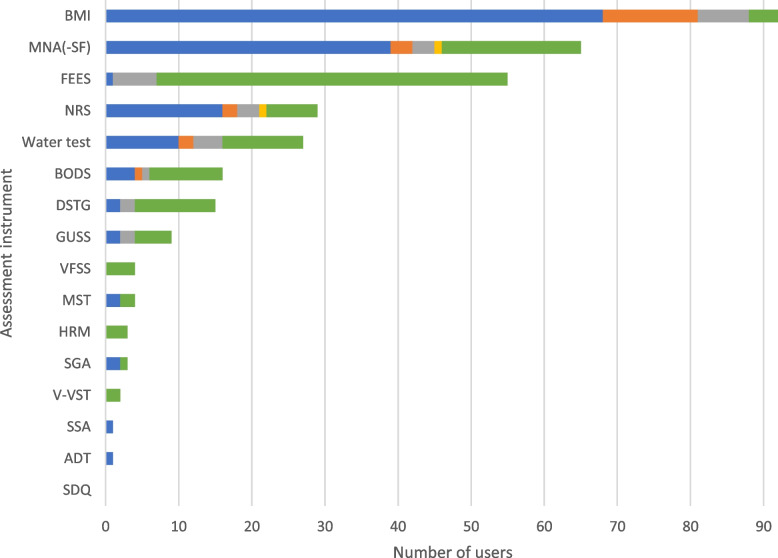
Dysphagia and nutrition assessment instruments. The use of the assessment instruments at the color-coded time points is presented in absolute numbers (blue = standardized on admission; orange = standardized before discharge; gray = standardized during inpatient treatment; yellow = standardized as post/progression/follow-up after inpatient treatment or on readmission; green = in the context of specific treatments/diagnoses). Multiple responses were possible. ADT = *Aachener Dysphagie Test*, BMI = Body Mass Index, BODS = *Bogenhausener Dysphagiescore*, DSTG = Dysphagie Screening Tool Geriatrie, FEES = Fiberoptic endoscopic evaluation of swallowing, GUSS = Gugging Swallowing Screen, HRM = High-Resolution-Manometrie, MNA(-SF) = Mini Nutritional Assessment (Short Form), MST = Malnutrition Screening Tool, NRS = Nutritional Risk Screening, SDQ = Swallowing Disturbance Questionaire, SGA = Subjective global Assessment, SSA = standardized swallowing assessment, VFSS = Videofluoroscopy, V-VST = Volume-Viscosity Swallowing Test

Almost all participants reported collecting a nutritional status and dysphagia assessment (*n* = 75, 98.7%; Fig. 2). The body mass index (BMI) was most commonly used standardized on admission (*n* = 68, 90.7%) and less commonly used before discharge (*n* = 13, 17.3%). Other assessment instruments frequently used on admission for nutritional status included the Mini Nutritional Assessment (-Short Form) (MNA(-SF); [37]) (*n* = 39, 52.0%), or the Nutritional Risk Screening (NRS; [38]) (*n* = 16, 21.3%). If dysphagia is clinically suspected, fiberoptic endoscopic evaluation of swallowing (FEES) was commonly used (specific situations: *n* = 48, 64.0%). Results are presented in Fig. 8.

### Social status and comorbidity

**Fig. 9:**
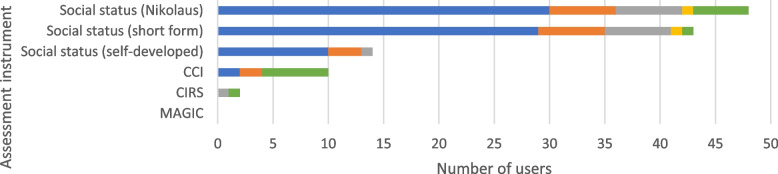
Social status and comorbidity assessment instruments. The use of the assessment instruments at the color-coded time points is presented in absolute numbers (blue = standardized on admission; orange = standardized before discharge; gray = standardized during inpatient treatment; yellow = standardized as post/progression/follow-up after inpatient treatment or on readmission; green = in the context of specific treatments/diagnoses). Multiple responses were possible. CCI = Charlson Comorbidity Index, CIRS = Cumulative Illness Rating Scale, MAGIC = Manageable geriatric assessment

The majority of respondents (*n* = 71, 93.4%; Fig. 2) reported recording social status or comorbidities in a standardized manner. In particular, two assessment instruments were used to record the social status on admission: The Nikolaus social status [39] (*n* = 30, 42.3%) and the short form social status (*Sozialstatus Kurzform*, *n* = 29, 40.8%). A self-developed social status scale was less frequently used on admission (*n* = 10, 14.1%). Scales assessing comorbidities such as the Charlson Comorbidity Index (CCI; [40]) (total: *n* = 6, 8.5%) or the Cumulative Illness Rating Scale (CIRS; [41]) (total: *n* = 1, 1.4%) were rarely used, mostly in specific situations. Results are presented in Fig. 9.

### Pressure ulcers

**Fig. 10:**
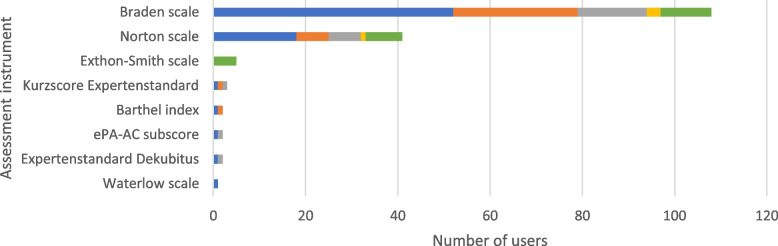
Pressure ulcers assessment instruments. The use of the assessment instruments at the color-coded time points is presented in absolute numbers (blue = standardized on admission; orange = standardized before discharge; gray = standardized during inpatient treatment; yellow = standardized as post/progression/follow-up after inpatient treatment or on readmission; green = in the context of specific treatments/diagnoses). Multiple responses were possible. ePA-AC = *ergebnisorientiertes PflegeAssessment Acute Care©*

The majority of the participants (*n* = 68, 89.5%; Fig. 2) reported regularly using assessment instruments for pressure ulcers. Of the eight scales listed for the assessment of pressure ulcers, two scales were used regularly: The Braden scale [42] and the Norton scale [43]. Both scales were used most frequently at admission (Braden: *n* = 52, 76.5%; Norton: *n* = 18, 26.5%) and less frequently before discharge (Braden: *n* = 27, 39.7%; Norton: *n* = 7, 10.3%). Results are presented in Fig. 10.

### Language and speech

**Fig. 11:**
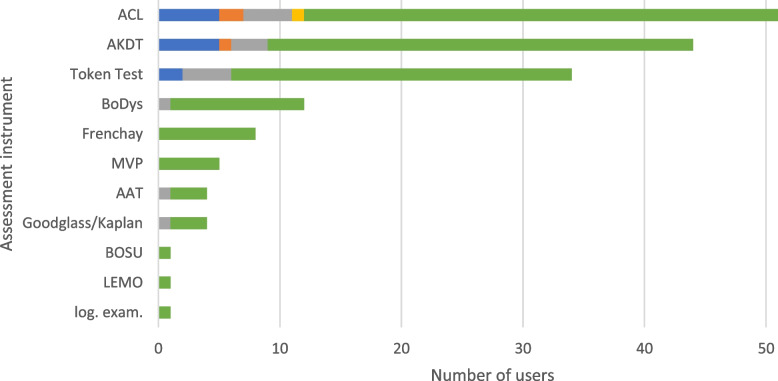
Language and speech assessment instruments. The use of the assessment instruments at the color-coded time points is presented in absolute numbers (blue = standardized on admission; orange = standardized before discharge; gray = standardized during inpatient treatment; yellow = standardized as post/progression/follow-up after inpatient treatment or on readmission; green = in the context of specific treatments/diagnoses). Multiple responses were possible. AAT = *Aachener Aphasie Test*, ACL = *Aphasie-Checklist*, AKDT = *Aphasie/kognitive Dysphasie-Testung*, BoDys = *Bogenhausener Dysarthrieskalen*, BOSU = *Bogenhausener Semantikuntersuchung*, Goodglass/Kaplan = *Kommunikationsskala nach Goodglass und Kaplan*, LEMO = *Lexikon Modellorientiert*, log. exam. = logopedic examination, MVP = *Münchner Verständlichkeitsprofil* (Munich Intelligibility Profile)

Language and speech assessments were performed by the majority of participants (*n* = 66, 86.8%; Fig. 2), most commonly within the indication of specific diagnoses/treatment. The most frequently used instruments in this context were the *Aphasie-Checkliste* (ACL; [24]) (*n* = 39, 59.1%), the *Aphasie/kognitive Dysphasie-Testung* (*n* = 35, 53.0%), the Token Test (*n* = 28, 42.4%) and the *Bogenhausener Dysarthrieskalen* (BoDys; [44]) (*n* = 11, 16.7%). Results are presented in Fig. 11.

### Delirium

**Fig. 12:**
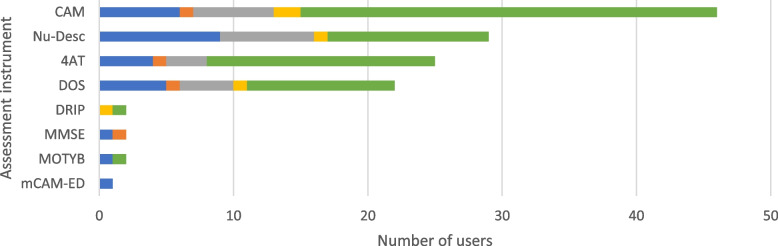
Delirium assessment instruments. The use of the assessment instruments at the color-coded time points is presented in absolute numbers (blue = standardized on admission; orange = standardized before discharge; gray = standardized during inpatient treatment; yellow = standardized as post/progression/follow-up after inpatient treatment or on readmission; green = in the context of specific treatments/diagnoses). Multiple responses were possible. CAM = Confusion Assessment Method, DOS = Delirium Observation Scale, DRIP = Delirium-Restricted Mobility Infection & Inflammation Psychosomatic, mCAM-ED = modified Confusion Assessment Method for the Emergency Department, MMSE = Mini Mental State Examination, MOTYB = Months-of-The-Year-Backwards, Nu-DESC = Nursing Delirium Screening Scale

Sixty-four of the 76 participants (84.2%; Fig. 2) reported that they perform a standardized delirium assessment. Delirium assessment instruments were most often used for specific issues, with the Confusion Assessment Method (CAM; [45]) (*n* = 31, 48.4%) being used most often, followed by the 4AT [46] (*n* = 16, 25.0%), the Nursing Delirium Screening Scale (Nu-DESC; [47]) (*n* = 12, 18.8%) and the Delirium observation Screening Scale (DOS; [48]) (*n* = 11, 17,2%). Results are presented in Fig. 12.

### Frailty

**Fig. 13:**
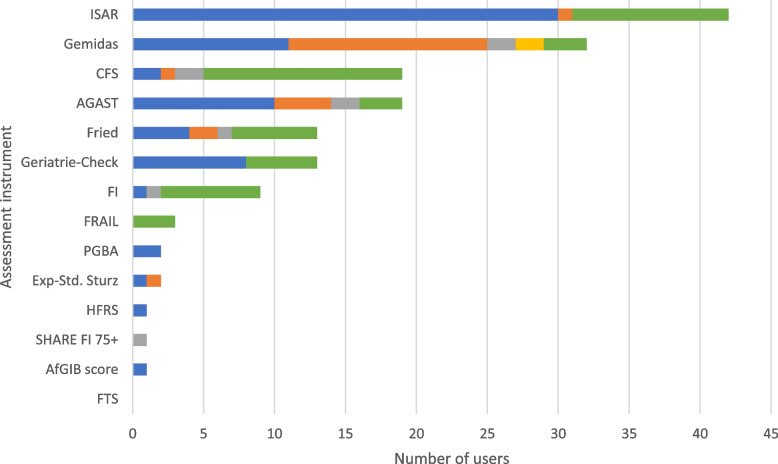
Frailty assessment instruments. The use of the assessment instruments at the color-coded time points is presented in absolute numbers (blue = standardized on admission; orange = standardized before discharge; gray = standardized during inpatient treatment; yellow = standardized as post/progression/follow-up after inpatient treatment or on readmission; green = in the context of specific treatments/diagnoses). Multiple responses were possible. AfGIB = Ärztliche *Arbeitsgemeinschaft zur Förderung der Geriatrie in Bayern*, AGAST = *Arbeitsgemeinschaft Geriatrisches Basisassessment*, CFS = Clinical Frailty Scale, Exp.-Std. = *Expertenstandard*, FI = Frailty Index, Fried = Cardiovascular Health Study Frailty Screening Measure, FTS = Frailty Trait Scale, Gemidas = *Geriatrisches Minimum Data Set*, HFRS = Hospital Frailty Risk Score, ISAR = Identification of Seniors at Risk, PGBA = *Pflegegesetzadaptiertes Geriatrisches Basisassessment*, SHARE-FI = Frailty Instrument for Primary Care of the Survey of Health, Ageing and Retirement in Europe

Sixty-four of the 76 participants (84.2%; Fig. 2) reported to regularly use frailty assessment instruments. The assessment instrument most frequently used was the Identification of Seniors at Risk (ISAR; [49]) (admission: *n* = 30, 46.9%; specific situations: *n* = 11, 17.2%). Other scales frequently used at time of admission were the *Geriatrisches Minimum Data Set* (Gemidas; [50]) (*n* = 11, 17.2%), the *Arbeitsgemeinschaft Geriatrisches Basisassessment* (AGAST; [51]) (*n* = 10, 15.6%) and the *Identifikation des geriatrischen Patienten* (*Geriatrie-Check*; [52]) (*n* = 8, 12.5%). Other scales were used more frequently in context of specific diagnoses/treatment, e.g. the Clinical Frailty Scale (CFS; [53]) (*n* = 14, 21.9%), the Frailty Index (FI; [54]) (*n* = 7, 10.9%), the Cardiovascular Health Study (CHS) Frailty Screening Measure (according to Fried) [55] (*n* = 6, 9.4%), or the FRAIL scale [56] (*n* = 3, 4.7%). Results are presented in Fig. 13.

## Discussion

The results of this survey indicate that 76 geriatric departments throughout Germany participating in this survey use standardized assessment instruments for motor function, self-help capability, cognition, depression and pain. The most frequently used instruments in these categories were the TUG, the BI, the MMSE, the GDS, and the VAS, respectively. When reviewing the S1 guideline for level 2 CGA, it becomes apparent that many assessment instruments recommended in the guideline are also used by the participants of the study. However, in some cases, the current implementation of the CGA is not consistent with the recommendations of the S1 guideline for level 2 CGA, while for some geriatric syndromes no standard operating procedures exist at all.

### Motor function

In the assessment of motor function, various parameters such as strength, walking speed, balance and transfer are being analysed. The TUG [13] is the most commonly used instrument to assess motor function. Following the S1 guideline for level 2 CGA [4], the TUG is particularly recommended as a screening instrument. However, since the results are abnormal in most geriatric patients, other instruments should be used subsequently, such as the Short Physical Performance Battery (SPPB) [57]. The SPPB is highlighted in the S1 guideline for level 2 CGA because of its high predictive value for adverse health outcomes such as falls [58], hospitalization [59] and mortality [60]. Our results suggest that the SPPB is currently rarely used in the geriatric setting in Germany.

The second most frequently used instrument among our participants is the Tinetti test [14]. This test is only conditionally recommended by the S1 guideline for level 2 CGA, since it places high demands on the examiner and requires the patient’s ability to get up. In many centers, grip strength is also measured in a standardized way. This is in line with the recommendations of the S1 guideline for level 2 CGA, as grip strength is simple to measure and has a close association with total body strength [61, 62]. Combined with the chair rise test, which was also frequently reported as an assessment instrument for motor function in the survey, grip strength is recommended for detecting sarcopenia [63]. The ETS [15] is recommended by the S1 guideline for level 2 CGA, as it assesses mobility at bed level or when transferring from bed to (wheel)chair and therefore enables differentiated mobility assessment of non-ambulatory patients. Our results show that it is used in a standardized way at different time points in many centers. The DEMMI is also recommended by the S1 guideline for level 2 CGA and is suitable for a differentiated assessment of mobility, also in non-ambulatory patients, as it comprises numerous tasks that do not require walking ability and has almost no floor effects [64]. Particularly for patients with previous falls, the S1 guideline for level 2 CGA recommends the use of assessment instruments for fear of falling, such as the FES-I [65], which has been used very rarely so far in the centers surveyed.

In summary, for the assessment of motor function, a combination of grip strength, TUG and SPPB (or ETS/DEMMI for non-ambulatory patients) can be recommended, noting that the SPPB should be used more frequently than it is currently the case.

### Self-help capability

The assessment of self-help capability comprises the completion of daily activities (e.g. bathing, dressing) as well as the ability to perform fine motor activities relevant to everyday life (e.g. money counting). In the survey, the BI [18] was most frequently used to assess self-help capability and was collected by almost all centers on patient admission. This is in line with the S1 guideline for level 2 CGA [4], which recommends the BI for recording self-help capability. As a possible supplement, the S1 guideline for level 2 CGA [4] suggests the IADL scale according to Lawton & Brody [22] to assess further activities such as household tasks, which are not covered by the BI. According to the survey, the IADL scale is currently used only rarely and mainly in the context of specific diagnoses and treatments. In contrast, the ADL scale [19] is more frequently assessed, which, in contrast to the IADL scale, covers the same activities as the Barthel index and therefore does not offer any additional information. Furthermore, it is important to consider that the survey answers include some instruments that, while not substitutes, provide added value to the BI and could therefore be used more frequently in the context of specific treatments or diagnoses. One example is the TTMC [21], as it screens for impairment in cognitive, fine motor, and sensory abilities, which may be related to reduced self-help capability.

In conclusion, the BI is recommended as a screening instrument, most preferably in combination with the IADL scale.

### Cognition

Cognition screening is mostly performed using assessment instruments that include the parameters of memory, attention, language, orientation, and executive functions (with the exemption of the CDT). Currently, the most commonly used CGA instrument to assess cognition is the MMSE [23]. According to the S1 guideline for level 2 CGA [4], the MMSE is well suited in the area of moderate dementia, but is inferior to the MoCA [25], DemTect [66], and TFDD [27] in the domain of mild cognitive impairment (MCI) [66–68]. This suggests that the regular use of the MMSE as a screening instrument for cognitive deficits in patients without initial suspicion of dementia, according to our survey, should be critically questioned. The MoCA, DemTect, or TFDD might be more suitable as standardized screening procedures, e.g., on admission, but are currently used by most centers only in exceptional cases. Another commonly used instrument is the CDT [69, 70], which, according to the S3 dementia guideline [71], should only be used in addition to other screening methods. Our results indicate that most centers combine the MMSE and the CDT. The S1 guideline for level 2 CGA also suggests the six-item screener (SIS) [72] as a time efficient screening instrument on admission, which was not reported to be used by any center in the survey results. However, many centers use the CERAD-NAB [26] in the context of specific diagnoses and treatments, which seems reasonable, as the CERAD-NAB is a CGA level 3 test battery that should not be used as a standardized screening instrument, but only in cases of abnormal screening results and suspected dementia.

In summary, the MMSE is currently the most commonly used instrument to screen for cognition on geriatric wards. We would like to point out, however, that other screening instruments have proven to be more sensitive for MCI and are therefore likewise recommended by the S1 guideline for level 2 CGA (e.g., MoCA, DemTect). Therefore, these assessment instruments should be recommended in all geriatric patients to also detect mild stages of cognitive impairment.

### Depression

There are a number of instruments in the geriatric field to assess mood and depressive symptoms such as sadness, hopelessness, and loss of interest. In the results of these analyses, the GDS [28] was found to be by far the most frequently used assessment instrument for depressive symptoms in the surveyed (neuro)geriatric centers in Germany. The S1 guideline for level 2 CGA [4] primarily recommends the short form of the GDS with 5 items (GDS-5) as a screening instrument in level 2a CGA in individuals without evidence of depression. However, it is pointed out that the WHO-5 [32] is more sensitive than the GDS in mild forms of depression [73, 74] and is therefore recommended as an assessment instrument by the S3 guideline for unipolar depressive episodes [75]. Therefore, we would like to point out that, in addition to the GDS, the WHO-5 should be used to detect depression especially when only mild depressive symptoms are being observed.

### Pain

A number of instruments are used for the assessment of pain in the geriatric setting, such as numeric or visual analogue scales, as well as more detailed questionnaires to assess parameters such as pain character, intensity, and frequency, and the impact of pain on coping with daily life. In our results, the VAS and NPRS were most commonly used for standardized assessment of pain. This is in line with the S1 guideline for level 2 CGA [4], which recommends the use of these instruments for patients without severe cognitive impairment. Alternatively, for patients with dementia, either the BESD [76] or the BISAD [33] should be used, which were shown to be frequently used in the context of specific diagnoses in our results. The S1 guideline for level 2 CGA indicates that in case of positive results in level 2 CGA, level 3 assessment instruments, such as the painDETECT [36], should be used. Although pain is a common symptom in geriatric patients, our results suggest that pain at level 2 CGA is assessed in accordance with guidelines, whereas at level 3 CGA, the recommended instruments are rarely used.

### Dysphagia and nutrition

To screen for malnutrition and dysphagia, questionnaires are being used that assess risk factors such as low BMI, weight loss, decreased mobility, or impaired cognitive abilities. Further assessment instruments include clinical swallowing examinations, such as water swallowing tests or more elaborate multiconsistency protocols and gold-standard diagnostic procedures, such as FEES. According to the S1 guideline for level 2 CGA [4], the recording of BMI is a basic requirement for the risk assessment of malnutrition. Therefore, it is assessed by almost all centers in a standardized manner on admission. Subsequently, the S1 guideline for level 2 CGA specifically recommends the MNA-SF [37] as a brief instrument to assess the nutritional status, which was also frequently cited in our survey. In addition, FEES was used by many centers in the context of specific diagnoses. In the S1 guideline for level 2 CGA, FEES is recommended as an instrument to improve diagnostic validity in cases of high-grade suspicion of dysphagia. The S1 guideline for level 2 CGA also states that although questionnaires for dysphagia have a high sensitivity, they are not suitable for planning the treatment regime, however, clinical and/or invasive examinations should be used as part of a stepwise diagnosis.

Based on our findings, we conclude that there is already widespread use of instruments to detect malnutrition and dysphagia in Germany. This seems to be in part due to the fact that logopedic assessment is a part of the multidisciplinary early rehabilitative geriatric treatment regimen. However, a considerable number of respondents indicated that the results of questionnaires or water swallow tests are used in the context of specific treatments and diagnoses. It should be noted that these procedures are screening tools intended to foster further diagnostic workup, but do not allow therapeutic conclusions to be drawn.

### Social status and comorbidity

The social status includes, among other components, the domestic situation, the social network, and actions which have been taken to provide current and future healthcare (e.g. nursing services, patient directive). Regarding the assessment of the social situation and comorbidities, the results of our survey do not provide a strong tendency regarding the application of the various assessment instruments. Overall, no instrument was regularly used by more than half of the centers. Most centers use the Nikolaus social status [39]. It should be noted that some centers have developed an individual questionnaire according to the most frequent social issues that are relevant for daily activities. Our ambiguous results are also reflected by the S1 guideline for level 2 CGA [4], which does not clearly recommend any instrument to record the social situation, because, so far, no instrument includes all elements required to capture the social situation in the setting of early rehabilitative geriatric treatment (housing situation, social contacts and activities, nursing support, legal dispositions). The same situation is found with the instruments for the assessment of comorbidities: instruments such as the CCI [40] or CIRS [41] are only applied by few centers. In summary, our results suggest a gap in the area of standardized and comparable recording of social status and comorbidities.

### Pressure ulcers

The instruments used to assess the risk of developing pressure ulcers include patient activity and mobility, incontinence, sensory function, age, weight, and cognitive ability. The Braden scale [42] was developed in 1987 and, consistent with the results of this study, is widely used for risk assessment for pressure ulcers in the inpatient setting. In a study of 642 hospitalized patients with heart failure, the Braden scale showed an association with 30-day mortality and length of hospital stay [77]. In comparison with other scales (Norton scale [43], Waterlow scale [78]) for risk assessment of pressure ulcers, a systematic review from 2006 found that the Braden scale had the best balance between sensitivity and specificity [79]. In another prospective study in the rehabilitation setting, the Braden scale achieved better specificity and positive predictive values than the Norton scale with similar good sensitivity values [80]. Nevertheless, there are doubts in the literature whether the formal recording of risk assessments compared with the clinical assessment of the nurse results in a reduced incidence of pressure ulcers [81, 82]. So far, there are no recommendations on the use of pressure ulcer assessment instruments in the S1 guideline for level 2 CGA [4].

### Language and speech

Language and speech disorders in geriatric patients have different causes such as vascular diseases (e.g. stroke) or neurodegenerative diseases (e.g. dementia) [24]. Screening should detect patients with speech disorders. Moreover, it should also be able to differentiate between deficits of language and cognition. The ACL [24] is most commonly used assessment instrument according to our results; it is a relatively new instrument developed in Germany, which can be used for aphasias of all causes and can distinguish cognitive dysphasia from aphasia by using nonverbal cognitive tasks. Nevertheless, it is evident from the results of our survey that instruments to assess language and speech are used almost exclusively in the context of specific diagnoses and therapies, and rarely in a standardized way. This may be due to the fact that there is a variety of assessment instruments and no clear recommendations for their use in, for example, post-stroke patients [83] or patients with dementia [84]. The S1 guideline for level 2 CGA [4] also does not include recommendations for the assessment of language and speech. Since deficits of language and speech have serious effects on the life of the affected patients, for example on quality of life [85] and psychological well-being [86], it is of great importance to improve the standardized diagnostics of language and speech disorders, for example by the regular use of screening instruments.

### Delirium

Assessment instruments for delirium include items such as orientation, communication skills, vigilance, and misperceptions (e.g., visual hallucinations). The S1 guideline for level 2 CGA [4] presents three assessment instruments for detecting delirium: The NuDesc [47], the DOS [48], and the CAM [45]. In particular, the NuDesc is pointed out as it is useful for earlier and more sensitive detection of delirium in the inpatient stay. While the NuDesc and the DOS are relatively short to perform and feasible instruments to objectify the risk of delirium, the CAM represents a more sophisticated and time-consuming diagnostic procedure and may be more suitable as a second instrument in stepwise diagnostics.

This stepwise diagnostic procedure is reflected in the results of our analyses as the NuDesc is more often performed in a standardized manner on admission than the CAM, and the CAM, on the other hand, is performed in the context of specific treatments or diagnoses. Nevertheless, it should be noted that only a minority of the surveyed centers use standardized screening instruments to detect delirium. This is astonishing as delirium has a high incidence (up to 50%) [87] in hospitalized geriatric patients and is associated with higher mortality, longer hospital stays and worse prognosis [88–90]. Although delirium prevention measures such as regular screening can prevent complications and improve patient prognosis [91], other studies have also found a lack of standardized approaches to delirium management [92, 93]. Reasons include lack of time and staff, as well as a lack of knowledge about delirium and its management, for example, the choice of assessment tools and when to use them [92, 93]. Another aspect that may contribute to the relatively low percentage of usage of standardized instruments for delirium screening is that participants' experience might be used instead of validated assessment instruments for identifying patients at risk for delirium. In summary, there is high potential for better detection and prevention of delirium on geriatric wards.

### Frailty

The frailty physical phenotype is a well-studied geriatric syndrome associated with decreased physical integrity and increased vulnerability to external stressors, resulting in an increased risk for adverse health events [94]. Screening and assessment instruments to capture frailty include mainly physical items such as strength, walking speed, weight loss, or need for assistance. In our survey, some clinicians reported to use instruments developed to screen for physical frailty risk (e.g. the ISAR [49], the CFS [53], the CHS Frailty Screening Measure (according to Fried) [55], the FI [54], or the FRAIL scale [56]), whereas other clinicians reported assessing frailty in the context of standardized geriatric assessment (e.g., Gemidas [50] or AGAST [51]). These results are consistent with an international survey [95] in which instruments assessing mobility, for example, walking speed or the SPPB, were used as frequently, or in some cases more frequently than the specific physical phenotype assessment instruments. Based on the results of this study, the ISAR as a screening instrument for physical frailty risk is most commonly used on geriatric wards in Germany, although other assessment instruments such as the CFS, the CHS Frailty Screening Measure (according to Fried), or the FI are found to be more robust [94, 96]. While the CFS or the CHS Frailty Screening Measure (according to Fried) are more suitable as screening instruments to identify predominantly physical frailty phenotypes, the FI is a more detailed measurement instrument to further classify frailty [97]. Therefore, in stepwise diagnostics, a shorter instrument should be used first to identify frail patients (e.g., CFS, CHS), followed by a more sophisticated instrument, such as the FI, to determine the frailty severity level [94, 97, 98]. Recently, also in light of the recognition of frailty as a multidimensional condition beyond the physical phenotype, CGA-based instruments are under development. Among these, the Multidimensional Prognostic Index (MPI), performed by geriatricians, shows the highest clinimetric properties and good feasibility also in non-geriatric settings [99–101].

The results of this study impressively illustrate the urgent need for a standard operating procedure to distinguish screening and assessment of frailty in its physical phenotype as a crucial geriatric syndrome from CGA-based assessments of multidimensional frailty as a surrogate marker of biological age [102].

### Limitations

First of all, it should be mentioned that our results cannot be generalized to all geriatrics wards in Germany, as the number of participants in the survey does not match the number of geriatric wards in Germany. Even more, due to the national differences in assessment instruments, conclusions about CGA on an international level should be drawn with caution. Nevertheless, to the best of our knowledge, this is currently the largest survey of its type in Germany and the number of participants enables us to identify trends. Furthermore, it is important to be aware that a pre-selection of geriatric assessment instruments was made for each category with expert consensus. An influence by the pre-selection cannot be ruled out, even though participants were able to enter additional assessment instruments.

## Conclusions

The most commonly used assessment instruments at German geriatric wards to capture motor function, self-help capability, cognition, depression, pain, and dysphagia and nutrition are recommended by the S1 guideline for level 2 CGA. Assessment instruments that should be used more frequently are the SPPB to assess motor function, the MoCA or DemTect to assess cognition and especially MCI, and the WHO-5 to assess primarily mild depressive symptoms. To further evaluate pain as a frequent geriatric symptom, level 3 assessment instruments should be increasingly used. For the assessment of delirium, the recommendations of the S1 guideline for level 2 CGA show conformity with the current usage, but the assessment instruments are often only used in the context of suspected delirium and rarely in a standardized manner. For the assessment of social status and comorbidities as well as language and speech, frailty, and pressure ulcers, there are no clear recommendations in the S1 guideline for level 2 CGA so far. This work reflects the current state of literature especially regarding research in the areas of pressure ulcers and frailty. Particularly for frailty, it is important to develop a standard operating procedure for assessment in geriatric wards. For the assessment of social status and language and speech, further development of assessment instruments and studies on their suitability in the geriatric setting are needed.

## Data Availability

The datasets used and/or analysed during the current study are available from the corresponding author on reasonable request.

## Abbreviations

ACL: *Aphasie Checklist*
ADL: Activities of daily living
AGAST: *Arbeitsgemeinschaft Geriatrisches Basisassessment*
BDI: Beck Depression Inventory
BI: Barthel index
BISAD: *Beobachtungsinstrument für das Schmerzassessment bei alten Menschen mit Demenz*
BMI: Body Mass Index
BoDys: *Bogenhausener Dysarthrieskalen*
CAM: Confusion Assessment Method
CCI: Charlson Comorbidity Index
CDT: Clock Drawing Test
CERAD-NAB: Consortium to Establish a Registry on Alzheimer’s Disease Neuropsychological Assessment Battery
CFS: Clinical Frailty Scale
CGA: Comprehensive Geriatric Assessment
CHS: Cardiovascular Health Study
CIRS: Cumulative Illness Rating Scale
DEMMI: De Morton Mobility Index
DGG: *Deutsche Gesellschaft für Geriatrie*
DIA-S: *Depression im Alter-Skala*
DOS: Delirium Observation Scale
ETS: *Esslinger Transferskala*
FAU: *Friedrich-Alexander-Universität Erlangen-Nürnberg*
FEES: Fiberoptic endoscopic evaluation of swallowing
FES-I: Falls Efficacy Scale – International
FI: Frailty Index
FPS: Faces Pain Scale
GDS: Geriatric Depression Scale
Gemidas: *Geriatrisches Minimum Data Set*
HADS: Hospital Anxiety and Depression Scale
IADL: Instrumental Activities of daily living
ICF: International Classification of Functioning, Disability and Health
ISAR: Identification of Seniors at Risk
MCI: Mild Cognitive Impairment
MMSE: Mini Mental State Examination
MNA-SF: Mini Nutritional Assessment—Short Form
MoCA: Montreal Cognitive Assessment
NPRS: Numeric Pain Rating Scale
NRS: Nutritional Risk Screening
Nu-DESC: Nursing Delirium Screening Scale
PAINAD: Pain Assessment in Advanced Dementia Scale
SIS: Six-item Screener
SPPB: Short Physical Performance Battery
TFDD: *Test zur Früherkennung von Demenz mit Depressionsabgrenzung*
TTMC: Timed Test of Money Counting
TUG: Timed-up-and-go
VAS: Visual Analogue Scale
WHO-5: World Health Organization-Five Well-Being Index
